# Urban Malaria: Understanding its Epidemiology, Ecology, and Transmission across Seven Diverse ICEMR Network Sites

**DOI:** 10.4269/ajtmh.14-0834

**Published:** 2015-09-02

**Authors:** Mark L. Wilson, Donald J. Krogstad, Emmanuel Arinaitwe, Myriam Arevalo-Herrera, Laura Chery, Marcelo U. Ferreira, Daouda Ndiaye, Don P. Mathanga, Alex Eapen

**Affiliations:** Department of Epidemiology, School of Public Health, University of Michigan, Ann Arbor, Michigan; Department of Tropical Medicine, Tulane University School of Public Health and Tropical Medicine, New Orleans, Louisiana; Infectious Diseases Research Collaboration, Mulago Hospital Campus, Kampala, Uganda; Caucaseo Research Center/School of Health, Universidad del Valle, Cali, Colombia; Department of Chemistry, University of Washington, Seattle, Washington; Department of Parasitology, Institute of Biomedical Sciences, University of São Paulo, São Paulo, Brazil; University Cheikh Anta Diop, Dakar, Senegal; College of Medicine, University of Malawi, Blantyre, Malawi; National Institute of Malaria Research (Indian Council of Medical Research), National Institute of Epidemiology Campus, Tamil Nadu, India

## Abstract

A major public health question is whether urbanization will transform malaria from a rural to an urban disease. However, differences about definitions of urban settings, urban malaria, and whether malaria control should differ between rural and urban areas complicate both the analysis of available data and the development of intervention strategies. This report examines the approach of the International Centers of Excellence for Malaria Research (ICEMR) to urban malaria in Brazil, Colombia, India (Chennai and Goa), Malawi, Senegal, and Uganda. Its major theme is the need to determine whether cases diagnosed in urban areas were imported from surrounding rural areas or resulted from transmission within the urban area. If infections are being acquired within urban areas, malaria control measures must be targeted within those urban areas to be effective. Conversely, if malaria cases are being imported from rural areas, control measures must be directed at vectors, breeding sites, and infected humans in those rural areas. Similar interventions must be directed differently if infections were acquired within urban areas. The hypothesis underlying the ICEMR approach to urban malaria is that optimal control of urban malaria depends on accurate epidemiologic and entomologic information about transmission.

## Introduction

Although malaria is typically considered mainly a problem of the rural poor, this disease has been a concern in urban settings for centuries. Today, however, evidence suggests that economic development and various environmental changes during the twentieth century have reduced the incidence of malaria in urban contexts.^1^ In addition, improved housing, drainage of *Anopheles* breeding sites, household mosquito proofing, expanded personal protection, effective diagnosis and treatment, and other factors have contributed to the recent global decline in malaria incidence. Whether, where, and to what extent such improvements have affected urban dwellers is still being debated.^1^–^4^ Indeed, a simple PubMed search (August 17, 2014) for research articles with titles containing “urban(ization)” and “malaria” yielded more than 200 publications during the past three decades that specifically focused on malaria among urban residents. These reports studied malaria across diverse urban settings of Africa, South America, and Asia and addressed the various factors that influence the risks of infection and disease in urban locations.

Among the concerns that complicate our analysis and understanding of “urban malaria” are 1) the definitions of what constitutes urban and urban malaria; 2) the accuracy of diagnosis by microscopy, rapid diagnostic tests (RDTs), and polymerase chain reaction (PCR)–based amplification of parasite DNA; 3) epidemiologic studies to localize transmission, including the assessment of potential confounders such as travel and the spatial heterogeneity of transmission; 4) entomologic studies to localize transmission and assess the intensity of transmission; and 5) methods to assess the completeness of surveillance and reporting.

**Figure 1.**
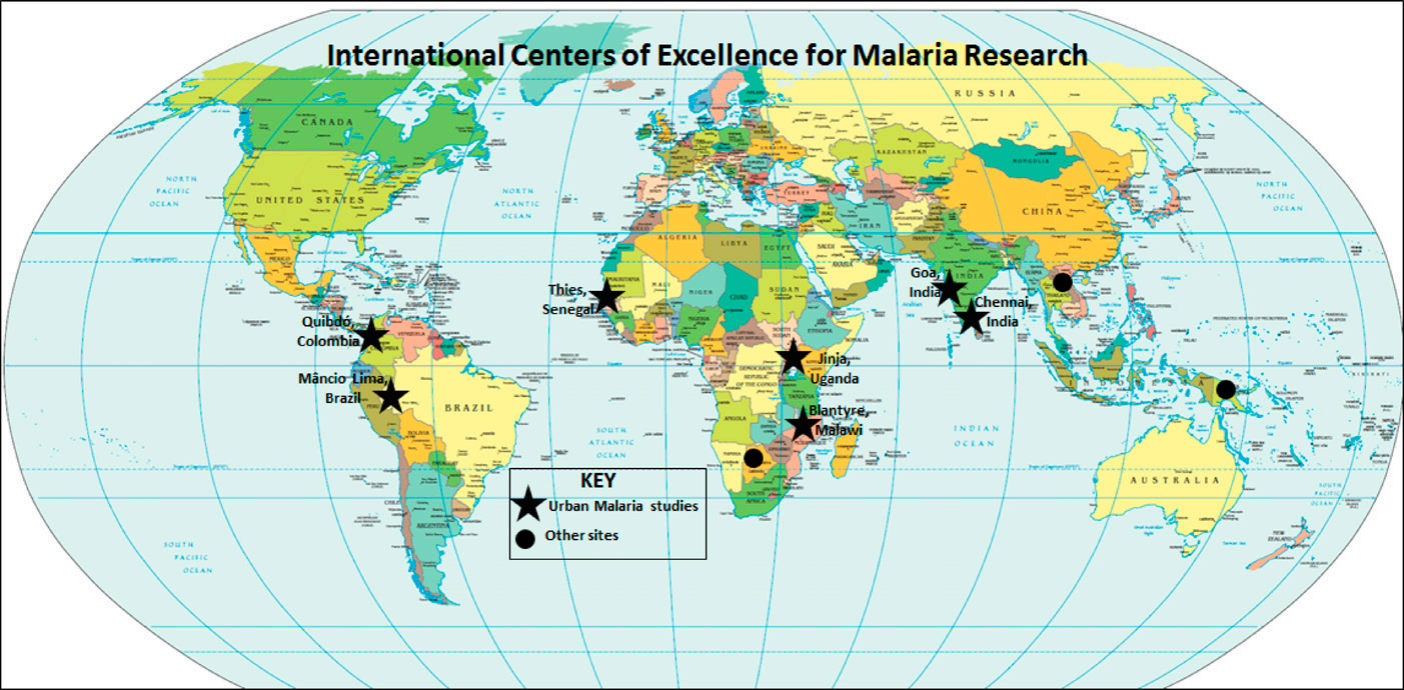
Locations of the International Centers of Excellence for Malaria Research (ICEMR) sites, including the seven sites reporting studies on urban malaria.

In an effort to better understand these issues in urban contexts, research is underway at locations supported by the National Institute of Health–funded International Centers of Excellence for Malaria Research (ICEMR) (Figure 1

). This report summarizes the questions that urban malaria research is addressing and characterizes the approaches, preliminary findings, and challenges faced by ICEMR sites across the globe. In this introduction, we present many challenges of defining and studying urban malaria to highlight issues that should be considered in evaluating patterns. We recognize that not all ICEMRs are gathering data to address each of these issues, but most have been collecting or will acquire data on some of the factors. By highlighting unresolved methodological issues, we provide examples of how the ICEMRs are attempting to address them in both similar and different ways.

Infectious disease risks in urban areas differ from those in rural settings, with urbanization having changed health patterns in those areas.^3^,^5^–^11^ Most of the studies that have identified differences between “urban” and “rural” environments in exposure risks and prevention opportunities recognize that those categories are poorly defined, differentially applied, and difficult to generalize. Despite problems with the definition of what constitutes urban, most urban settings in sub-Saharan Africa (SSA) are now viewed as presenting an increased risk of various infectious diseases. In particular, the importance of malaria in urban contexts has recently been recognized,^12^ even if it is not well understood. The burden of malaria includes not only the loss of life and productivity, but the costs of prevention and treatment that have historically been viewed primarily as problems in rural settings. Similarly, some studies have suggested lower risk in urban areas compared with neighboring rural areas,^3^,^4^,^13^,^14^ although the unresolved question of what constitutes an urban versus a rural area makes comparisons difficult. Indeed, there is an enormous diversity of ecologies, exposure patterns, and prevention opportunities at different locations, making simple claims about urban versus rural risk very doubtful. Regardless, what is clear from many studies and a few meta-analyses is that the number of malaria cases in some urban settings is considerable, due in part to the increasing number of people who are becoming residents of urban environments.

### Defining urban.

No consensus or commonly accepted definition of urban has been used by international bodies, national or local governments, planners or policy makers, let alone by health scientists.^7^,^15^ United Nations Population Division data for 228 countries indicate that 108 use administrative definitions (e.g., city resident) to define urban, 51 use population density (e.g., people per square kilometer), and 39 use functional characteristics (e.g., extent of nonagricultural economic activity), whereas no definition exists for 22 and 8 classify either all or none of their citizens as urban.^3^ Thus, because each national Demographic and Health Survey relies on that country's National Statistical Office for their definition of urban, it is difficult to compare with other countries because of such differences. Urban communities have been defined by characteristics such as population number, density within specific geographic areas, or ensembles of people with governance responsibilities. For example, a settlement in Uganda with more than 100 people is classified as urban, while urban locales in Nigeria are those with more than 20,000 inhabitants.^16^ Alternatively, settings in Ethiopia with at least 2,000 people are classified as urban, whereas in Botswana urban areas include agglomerations of at least 5,000 inhabitants, most of whom depend on nonagricultural activities.^17^ These examples are all from SSA. However, similar lack of clarity and consistency is seen in Latin America, Asia, and other regions. In addition, efforts to quantify or compare urban and rural malaria have begun to explore various complex metrics that view this false dichotomy as a continuum.^18^

A related issue involves the spatial heterogeneity of conditions within an urban boundary. Particularly in poor but rapidly developing urban areas, important spatial variation in urban features may be missed if the average or modal conditions are assumed to represent the entire setting. Consider how crowded urban slums, open city parks, dense commercial centers, or “suburban” fringe residences are often interwoven within the boundary of what is considered urban. Thus, spatial heterogeneity, hence scale, add to the confusion of what constitutes risk.^19^

Nevertheless, investigators often agree on a qualitative, if subjective, definition of urban as an area with a sufficient density of housing and population that the setting is not a rural environment comprising open land with many fewer people and dwellings. However, to carefully evaluate and compare different areas, a more quantitative definition of urban is needed. One example is that of Phillips.^20^

… a geographic region whose boundaries are specified by a municipal/national government authority; which contains one or more areas with a high concentration of businesses, housing, paved streets and roads; with a high population density; where agriculture is regulated by a municipal authority; and with a total population size that exceeds 15,000 people.

Although it may be intuitively rational, this definition illustrates some of the challenges to characterizing urban malaria risk, because it does not consider the area of land (hence human population density), the extent of infrastructure concentration, or the type and extent of crop production. These and other features of the built environment are critical to many features that contribute to malaria risk, some of which can be detected using remotely sensed (satellite image) data.^21^,^22^

### Defining urban malaria.

The extent of malaria within spatial subunits can be described, but only after the definition of what constitutes malaria is clarified. Is the appropriate metric one of infection, disease, or both? What level of accuracy is needed based on the goals of surveillance? How is temporal variation (seasonality) recognized? These and other considerations will affect the presence and intensity of malaria incidence or prevalence in urban settings. Dissimilar definitions and diverse metrics make those comparisons difficult or impossible.

Defining urban malaria is also challenging because the same term is used to describe situations ranging from obviously imported cases (people infected in known endemic areas, who are then diagnosed after traveling to urban regions of non-endemic areas) to circumstances in which there is strong evidence for urban transmission in known endemic areas. In addition, substantial uncertainty often exists about the travel histories of infected people and the evidence for urban transmission within endemic areas. A better understanding of “imported” malaria is particularly important in urban settings, which constitute hubs of human mobility and migration. For those reasons, the guidance we have provided on this question (Table 1 ) emphasizes the effects of differences (gradients) in these parameters and the effects of those changes rather than a static (less flexible) classification.

### Defining “urban malaria transmission.”

Although solid evidence of human infection among urban residents is important, the critical need is to determine how much “local transmission” is ongoing in and around urban dwellings. Different kinds of observations represent stronger or weaker evidence that permits development of more or less robust inferences (Table 1). To help focus and design better interventions, it is essential to know whether people are being infected in the urban areas where infection and disease are being diagnosed. If not, then information on travel histories becomes critical to determining activities and locations of risk. For infections that are acquired in urban settings, it is then important to understand when and where transmission is occurring and how urban transmission may differ from that in rural settings. In particular, it is crucial to characterize and understand how urban microhabitats promote *Anopheles* vector abundance and influence their behavior of biting humans. Urban microclimate variables (e.g., temperature, relative humidity, and precipitation) are also crucial to mosquito survival, reproduction, and development, thereby influencing vector presence and abundance in urban environments.^23^ Beyond vector abundance, house construction, insecticide-treated net (ITN) use, and other factors that influence human–vector contact represent important factors in transmission risk. Similarly, an understanding of the amount of spatial variation that exists in urban areas with *Plasmodium* transmission and its magnitude are essential for knowing what kinds of control or prevention efforts should be undertaken in those settings.

A recent systematic review of factors contributing to *Plasmodium* transmission in urban areas of SSA^24^ evaluated more than 100 peer-reviewed studies from throughout the subcontinent. Most of these studies documented that cases were being steadily recorded in urban settings, at a surprisingly high incidence, although malaria incidence in urban areas was typically lower than in the surrounding rural settings. However, lower incidence in high population urban areas may represent a large number of urban malaria cases. Fewer studies have documented the vector-related characteristics (breeding sites, suitable environments, among others) found in these urban environments. Finally, only a few studies have provided direct evidence of urban transmission (vector feeding and vector infection), consistent with the difficulty of obtaining evidence for transmission in urban settings.

### Epidemiologic studies and localization of urban transmission.

Epidemiologic factors consistent with the presence and importance of focal transmission include spatial patterns of focal heterogeneity in the prevalence of human infection from cross-sectional surveys or the incidence of disease from passive case detection (PCD). Most PCD occurs through health facility–based summaries of malaria treatment, whether summarized in government reports or gathered from facilities by researchers. Active case detection (ACD), however, involves researchers intentionally testing people and identifying cases through research based in the community. Although a number of reports have noted that ACD is more sensitive than PCD,^25^–^27^ the greater cost of ACD in combination with modest budgets has limited most ICEMR investigators in SSA (except Malawi) to the use of PCD for the assessment of urban malaria. In Indian cities with high population densities, the lack of adequate manpower limits the use of ACD on a routine basis. Nevertheless, follow-up contact and confirmation microscopy for *Plasmodium falciparum* cases is undertaken to reduce further transmission.

Travel by residents of urban areas can put them at increased risk of both infection and disease and may thus limit the accuracy of inferences about urban transmission based on the diagnosis of malaria in urban areas. These may be short-term visits (days, weeks) to more rural areas of high risk, longer-term residency shifts (months), or more permanent migration (years). Malaria parasite infections acquired outside urban settings may be diagnosed subsequently in cities or towns where health facilities are more accessible and thus attributed incorrectly to urban transmission in the absence of diligent travel histories and entomologic studies. Careful sampling in heterogeneous urban neighborhoods is necessary to obtain representative survey results,^28^ and questionnaire-based travel histories can be essential to determine where urban residents actually became infected.^29^ More generally, travel is a potential risk factor for malaria among residents in both urban and rural settings.^30^–^33^

### Travel and changes in urban malaria.

Movement of a more permanent nature, in particular the movement of rural residents to urban and peri-urban areas (i.e., urbanization), is a major demographic trend with health impacts occurring in many parts of the globe.^19^ However, the effects of urbanization on *Plasmodium* transmission and malaria risk are not well understood. Decade-old reviews of studies addressing urbanization and malaria by Keiser and others,^11^ Tatem and Hay,^7^ Hay and others,^3^ and Utzinger and Keiser^6^ summarized various research projects where this issue had been investigated until then. In addition, recent work has added to our understanding of changing malaria risk in relation to migration in east Africa,^33^ as well as global patterns of disease caused by *Plasmodium vivax*.^35^ These studies and the work of Tatem and others^1^ suggest that the incidence of malaria tends to be lower in urban areas and that urbanization is accelerating as global malaria incidence is decreasing. However, because this concordance of increasing urbanization and decreasing malaria is occurring during a period of rapid scale-up of antimalarial interventions, it is difficult to ascribe causation or predict the future with confidence.

### Entomologic studies informing localization and intensity of transmission.

Entomologic factors consistent with the presence and importance of urban transmission include the presence of known *Anopheles* vectors in collections from pyrethrum spray catches (PSCs) and human landing catches (HLCs), evidence for human blood meals in blood-fed anopheline vectors, sporozoite-infected (circumsporozoite surface protein [CSP]–positive) anopheline vectors and elevated nightly biting rates and entomologic inoculation rates (EIRs) (Table 1). However, it is often more difficult to obtain reproducible estimates of entomologic parameters in urban areas (possibly because of less intense transmission and lower vector densities) than in rural areas (which typically have more intense transmission). For example, the low numbers of vectors obtained from PSCs and HLCs in some urban areas are often associated with sporozoite prevalence of 0% (in part because of the Poisson distribution effects with small sample sizes) and thus with EIR estimates of 0.0 infective bites/person/month, although hundreds of malaria cases are being diagnosed each month in the same urban area(s). Despite these constraints of small sample sizes on EIR estimates, the presence of competent *Anopheles* vectors in urban settings where humans are infected is strongly suggestive of local transmission (Table 1).

Vector incrimination and estimation of the urban EIR^36^ are critical to evaluating how much infection of urban residents results from local urban transmission. As Robert and others summarized in 2003,^37^ the heterogeneity of EIRs across the urban-to-rural gradient, and even within individual urban settings, is considerable. In more recent studies, for example, two areas of downtown urban Dakar, Senegal, were evaluated for *Plasmodium* infection of *Anopheles gambiae* s.l. at the end of the rainy season, producing estimated annual EIRs ranging from 3 to 9.5.^38^ Similar studies in Libreville, Gabon, compared *Anopheles* abundance, feeding, and infection during rainy and dry seasons in five districts of the city, showing very heterogeneous patterns, with EIRs for *An. gambiae* s.l. ranging from 0 to 19.2 during the dry season and from 2.5 to 68.7 during the rainy season. Curiously, the highest EIRs in the Libreville study were found in the most central and urbanized area. In contrast, in the municipality of Kandi, Benin, Govoetchan and others^39^ demonstrated that while rural transmission by *An. gambiae* s.l. was much higher, EIRs of 7.5 PPY (per person per year) were observed in the urban areas during the rainy season. Still another study in Mbalmayo, Cameroon (population ∼65,000), demonstrated perennial transmission of *P. falciparum* by *An. gambiae* (84%), *Anopheles moucheti* (11%), and *Anopheles funestus* (5%) that produced an average annual EIR of 129 in that urban setting.^40^ Overall, transmission in urban areas is generally lower than in rural areas but can be surprisingly elevated in some urban environments.

## Research on Urban Malaria at ICEMR Network Sites

The varied ways in which urban malaria is being studied by seven ICEMRs throughout the globe demonstrate the challenges of characterizing and comparing patterns of infection and disease risk. Investigations at these seven sites are described briefly below, with summary information on the biophysical characteristics (Table 2), study design (Table 3), parasite and vector species (Table 4), and the major research questions being examined (Table 5).

### Amazonia ICEMR, Mâncio Lima City, Brazil.

Mâncio Lima City on the Moa River is the westernmost city in Brazil, has a low population density of 1.4–3.0 inhabitants/km^2^, an average daily temperature of 27°C and 2,200 mm (220 cm = 7.2 feet) of rain per year. With a 0.5–1.3% prevalence of *P. falciparum* infection (based on thick smears) and a 1.4–4.1% prevalence of *P. vivax* infection (based on thick smears), malaria is an important public health problem and a potentially serious obstacle to socioeconomic development. With distinct seasons (dry from July to September, wet from October to June) and limited entomologic information available, potential high priority questions to consider include the use of long-lasting insecticidal nets (LLINs) and the availability of free artemisinin combination therapy (ACT) treatment of persons who have uncomplicated malaria. Because interventions that interrupt transmission are likely to be the most effective, the most important question in terms of malaria control may be when vector-borne transmission and the prevalence of parasitemia begin to increase each year.

### East Africa ICEMR, Jinja City, Uganda.

Annual rainfall and the average daily temperature were lower in Jinja City, Uganda, than in either Quibdó City, Colombia, or Mâncio Lima City (1,334 versus 8,131 and 2,200 mm; 23°C versus 27° C and 27° C, respectively). Nevertheless, the prevalence of *P. falciparum* infection was higher in Jinja City (8.8% versus 2.6% and 0.5–1.3%) and the EIR in Jinja City was substantial (2.8 infectious bites PPY [per person per year]) although no EIR data were available for comparison from Quibdó City or Mâncio Lima City. Preliminary results from Jinja City indicate that the malaria control strategies implemented have been associated with reductions in both the prevalence of infection (from 9.8% to 6.7%) and the EIR (from 4.0 to 1.8 PPY) and thus suggest that those interventions have been effective. The greater prevalence of infection in Jinja City than in Quibdó City or Mâncio Lima City indicates that other factors in addition to annual rainfall and average daily temperature also affect the prevalence of human infection in these malaria-endemic areas.

### India ICEMR, Besant Nagar Community in Chennai, India.

The city of Chennai has the highest population density of the seven ICEMR urban sites with 9,524 persons/km^2^ in the study site of Besant Nagar, substantial annual rainfall (1,400 mm/year), and the highest average daily temperature (33°C).^47^ Therefore, the prevalence of infection (parasitemia) in Besant Nagar (18.6%) was higher than in either Mâncio Lima City or Jinja City (0.5–1.3% and 8.8%, respectively). Chennai city accounts for 57–78% of all malaria cases recorded in the state of Tamil Nadu, with 93.6–99.7% of cases due to *P. vivax*. *Anopheles stephensi*, the vector responsible for transmission in the urban Chennai context, breeds in clean/clear stored water in overhead tanks, wells, cisterns, curing tanks/pits, underground tanks or sumps, earthen pots, roof gutters, and other such artificial containers.^23^

### Latin America ICEMR, Quibdó City, Colombia.

An important characteristic of the environment in Quibdó City on the banks of the Atrato River in Colombia is the overwhelming amount of rainfall each year in this tropical rain forest area (8,200 mm = 323 inches or 27 feet of rain per year). This means that 25 of every 30 days of each month are rainy days, with one continuous tropical rainy season throughout the year and an average daily temperature of 27°C. The prevalence of infection in the community (from preliminary studies based on positive blood smears for asexual *P. falciparum* parasites) is 2.6%. Paradoxically, the most intriguing question raised by the data from this urban site is “Why is the prevalence of parasitemia in the community only 2.6%? Why is it not higher?” Factors potentially relevant to this question include variation in the human population (sickle cell, glucose-6-phosphate dehydrogenase), in the vector (zoophilic versus anthropophilic biting behavior, extrinsic incubation periods and conditions), and in the parasite population (*var* gene expression, single nucleotide polymorphisms related to antimalarial resistance).

### Malawi ICEMR, Communities within Blantyre, Malawi.

The most striking feature of these data was the high population density within the urban communities studied in Blantyre, Malawi (consistent with extensive urbanization). Among the seven ICEMR urban sites, the population density in these urban sections of Blantyre (4,400 persons/km^2^) was exceeded only in Besant Nagar (10,988 persons/km^2^). Although total annual rainfall was similar (1,127 mm in Blantyre versus 1,400 mm in Chennai), the prevalence of infection was greater in Chennai than Blantyre (18.6% versus 5%, respectively, during the dry season and 8% in the rainy season). One of the unresolved questions raised by these data is why the prevalence of malaria in the urbanized areas of Blantyre (5–8%) is not closer to the similarly urbanized area of Chennai (19%). To confirm or reject candidate hypotheses, potential explanations (such as differences in the vector, parasite, or human populations) will need to be tested in additional communities at other sites with similar population densities.

### South Asia ICEMR, Panaji City in Goa, India.

As estimated from annual rainfall and average daily temperatures, environmental conditions in Panaji City, India, were basically similar to those in Chennai (2,932 and 1,605 mm annual rainfall, 27° and 29°C average daily temperatures, respectively). Goa is one of four sites being studied in the south Asia ICEMR and is potentially the most relevant for understanding urban malaria in south Asia. Goa is India's smallest state and is composed of contiguous urban centers including the capital city of Panaji.^64^ With a gross domestic product per capita 2.5 times that of the whole country and as much as 10 times greater than states such as Assam, Jharkhand, and Orissa, infrastructure projects in Goa attract migrant workers from the east and northeast of India.^65^–^67^ These states are rural and have the highest malaria endemicity in the country.^68^ In addition, tourism is Goa's largest industry, with many domestic and international tourists visiting year-round.^69^

One potentially important factor in the spread of malaria in India, particularly *P. falciparum*, is human migration. For south Asia malaria control experts, the link between human migration and the importation of malaria into urban settings is of enormous interest. Migrants may not have access to government health services and may therefore be exposed to preventive and treatment strategies different than national malaria control recommendations (e.g., use of ITNs and correct choice of antimalarial drug/drug regimens).^70^ However, the greatest worry is that migrants may facilitate the movement of drug resistance from northeast India to large urban centers in the west and southwest parts of India.^71^–^73^

The south Asia ICEMR is interested primarily in understanding how parasite populations vary in genetic plasticity across India and how that variation affects drug resistance, pathogenesis, and the ability of the parasite to overcome innate and acquired immunity. Those questions are now being addressed in part through passive surveillance at a public tertiary care hospital (Goa Medical College and Hospital), via vector collections in semi-enclosed living quarters at construction sites and with the aid of parasite collections in other states (Assam and Jharkhand).

### West Africa ICEMR, Community of Madina Fall in Thiès, Senegal.

The most striking feature of the data from Madina Fall is the discrepancy between the substantial numbers of cases diagnosed at the regional clinic for this area of Senegal (1,000–2,000 cases of uncomplicated *P. falciparum* malaria per year) and apparently contradictory factors such as the low prevalence of infection in the community (< 1% based on thick smears), the virtual absence of sporozoite-infected (CSP-positive) vectors, and—as a result—low or undetectable EIRs (0.0 infectious bites/person/month or year) at times when hundreds of cases are being diagnosed at a clinic within the same community each month.

The most interesting potential explanation for the greater numbers of malaria cases in Madina Fall than the surrounding urban areas is a gradient from primarily urban to primarily rural environments, which progresses from smaller to larger numbers of vectors and from lower to higher prevalences of human infection in association with active breeding sites in the most rural areas of Madina Fall where there is irrigation and vegetable farming. This hypothesis, if confirmed, suggests that malaria control strategies for such hot spots should focus on active breeding sites and should begin before the number of adult vectors and the prevalence of human infection begin to increase after the seasonal rains begin.

## Implications of ICEMR Analyses of Urban Malaria

The practical implications of better understanding the patterns and causes of *Plasmodium* infection and malarial disease in urban areas are many. Depending on how much transmission is occurring at the urban site versus elsewhere, prevention and control strategies may need to be modified extensively. For example, indoor residual spraying (IRS) will have no effect on malaria transmission if mosquitoes are not biting indoors. In India, vector control measures in urban settings are based primarily on larval source management, which is achieved by preventing ovipositioning by *An. stephensi* in artificial water storage containers. However, this approach becomes more difficult and less successful in other settings where natural, rain-fed bodies of water or irrigation channels serve as breeding sites. In those settings, additional preventive strategies may include specific education to remind travelers to use ITNs, LLINs when away from their homes. As a result, the effectiveness of ITNs^74^ and of ITNs versus IRS^75^ may vary substantially in urban versus rural settings.

Similarly, treatment may be ineffective and inappropriate if antimalarial drugs are taken for malaria-like symptoms in the absence of a positive smear or RDT. Conversely, testing for malaria may be delayed or unavailable if it has been assumed that malaria is rare or nonexistent in urban areas.

The small-scale spatial heterogeneity in urban transmission associated with clusters of people living near parks, water bodies, small-scale agricultural land, or peri-urban fringes also makes prevention difficult if neighborhood-specific risks are not known or considered. For example, urban breeding sites for *An. gambiae* in SSA (e.g., small urban gardens) and *An. stephensi* in India (e.g., urban water storage tanks) are microhabitats that are often unrecognized in large urban areas. This observation highlights the importance of identifying “hot spots” of transmission^76^ that require intervention at the local level. In addition, the generally lower frequency of malaria in urban settings also creates challenges for surveillance. This is because the lower prevalences of infection and lower incidences of disease in those areas mean that foci of transmission are more difficult to detect. As suggested during a recent study of urban malaria in Ouagadougou, Burkina Faso,^48^ irregularly or sparsely constructed dwellings near irrigation networks are locations where preventive strategies focused on urban children can be very effective.

The frequency of *Plasmodium* infection and the frequency and severity of malarial disease among urban residents vary by ICEMR regional epidemiologies and vector ecologies. The goal of these studies is to identify patterns across the urban sites within the ICEMR network that can be used to improve malaria control. However, in each case, detailed knowledge about local conditions will likewise be essential to reduce the intensity of urban transmission. We hypothesize that the urban sites that succeed—those that markedly reduce the frequency of both malarial infection and disease—will be the sites that most clearly define the ways in which transmission at their site is similar to and different from urban transmission at other urban ICEMR sites.

## Figures and Tables

**Table 1:** Types of evidence used to infer whether urban malaria is the result of transmission in an urban setting

Strength of evidence	Human surveillance	Human surveillance metrics	Entomological surveillance	Entomological indices	Parasite or vector genetic analysis
Weakest	Febrile disease at urban clinic	–	–	–	Parasite count (no. per microliter blood)
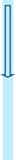	Slide or PCR confirmed diagnosis at urban clinic	PP of humans that are thick smear positive	Nearby presumed breeding sites	–	Infected humans: MOI from MSP1 genotyping, molecular barcodes, microsatellites, whole genome sequencing, SNP arrays
	Confirmed infection in UR	Incidence of laboratory confirmed malaria (*N*)	CV larvae/pupae in breeding site (*N*)	–	–
	More infection in permanent URs than in travelers	PP higher in “urban” than “rural” setting; PP higher in permanent URs than travelers	CV adults in houses (vector density/immature density); cohabitation with other vectors in breeding sites	Human blood meal index (% blood-fed and ELISA positive)	Malaria parasites: frequencies of drug resistance markers such as *pfcrt*, *dhfr*, *dhps*, *pfmdr*, and *kelch 13* variants
	–	Higher incidence (*N*) in permanent URs than in travelers	Blood-fed CV adults resting inside house (may be endophilic or exophilic)	HBR	–
	Greater PP prevalence among non-travelers	Greater PP among “non-travelers”	Sporozoite-infected adult mosquitoes	SP (% of CSP-positive adult female CVs)	–
	–	–	HLC or bednet trapping of CV (*N*)	EIR estimate	Anopheline vectors: markers for M and S chromosomal forms, PCR for species confirmation, drug resistance markers (*kdr* and *ace-1*)
Strongest	Confirmed *Plasmodium* infection with no history of travel and *Anopheles* in house	ACD of elevated PP among sick and healthy people lacking travel history	Blood-fed gametocyte-infected adult mosquitoes and breeding sites nearby household	Peri-domestic breeding site(s) + sporozoite-infected adult mosquitoes + CSP-positive CVs	Similar or identical parasite clones in anopheline vectors and humans from same urban settings

ACD = active case detection; CSP = circumsporozoite protein; CV = competent vector species; EIR = entomological inoculation rate (infective bites/person/year); ELISA = enzyme-linked immunosorbent assay; HBR = human biting rate; HH = household(s); MOI = multiplicity or clonality of infection; MSP1 = merozoite surface protein-1; PCR = polymerase chain reaction; PP = parasite prevalence in humans; SNP = single nucleotide polymorphism; SP = sporozoite prevalence; UR = urban resident(s).

The strength of evidence for *Plasmodium* parasites, *Anopheles* vectors, and relationships with humans is ranked from weakest to strongest with regard to implicating local urban transmission.

**Table 2:** Summary of the geographic, demographic, and climatic characteristics of each of the seven ICEMR sites that are studying malaria in urban settings

ICEMR (urban site, region, country)	Amazonia (Mâncio Lima City, Acre State, Brazil)	East Africa (Jinja City, Jinja District, Uganda)	India (Besant Nagar, Chennai City Tamil Nadu State, India)	Latin America (Quibdó City, Chocó Department, Colombia)	Malawi (Communities in Blantyre City, Malawi)	South Asia (Panaji City, Goa State, India)	West Africa (Madina Fall, Thiès City, Thiès Region, Senegal)
Latitude/longitude	7.62° S, 72.89° W	0.45° N, 33.24° E	13.00° N, 80.27° E	5.69° N, 76.66° W	15.67° S, 34.97° E	15.50° N, 73.83° E	14.83° N, 16.92° E
Elevation (m)	200	1,140	6	30	766	7	60
Area (km^2^)	5,453	12,853	∼21	3,338 (6,078)	228	36	2.47
Population	16,795	31,900	4.7 M	120,000	1.01 M	114,405	320,000; 21,000 Madina Fall
Population density	2.79/km^2^	2.5/km^2^	10,988/km^2^; 0.2 M Besant Nagar	362,625; 36.0 (59.7)/km^2^	4,400/km^2^	3,177/km^2^	8,502/km^2^
Climate	Dry: July–September	Wet: March–May and September–November	Tropical monsoon	Transmission throughout year	Tropical highland	Hot humid: March–May	Sudan–Savanna
Seasonality	Wet: October–June	Dry: December–February and June–August	Hot dry (March–June); Hot Wet (Oct–Dec)		Mild dry (May–September); Hot wet (October–April),	Monsoon: June–September; Winter: December–February	Mild dry (January–June), Hot wet (July–November)
Rainfall (mm)	2,200	1,334	∼1,400	8,131 (≥ 500/month)	1,127	2,932	438
Mean temperature (°C)	27	23	33	27	22	27	24
Estimates of prevalence, intensity of transmission	Prevalence: *Pf* = 0.5–1.3%, *Pv* = 1.0–4.1%	Prevalence = 8.8% (7.5–10.2%); Incidence = 0.48 (0.42–0.55) PPY	Prevalence = 18.6% (2014); 77% prevalence decline at Besant Nagar (2011–2014)	Prevalence: *Pf* = 2.6%; 5,064 cases (2012) with 31% from urban areas	Year-round transmission; slide prevalences: ∼5% (dry season), ∼8% (rainy season)	Slide prevalence: *Pf* = 0.02–0.6%, *Pv* = 0.2–1.3%	Prevalence < 1%, incidence uncomp-mal ≤ 1.1 × 10^−3^/month, 1,000–2,000 dx/year at referral clinic
	EIR = unknown	EIR = 2.8 (1.6–4.5) PPY	EIR = unknown	EIR = unknown	EIR = unknown	EIR = 2.35 PPY	EIR = 0.0 PPY, 0.0% vectors CSP positive
Aims, questions, hypotheses	Determine if in active/abandoned aquaculture produces vector breeding sites that facilitates urban transmission	XS and entomological surveys, longitudinal cohort, capacity building, technology transfer, research-policy maker links	Evaluate vector transmission, drug resistance, sexual/asexual infection, mixed species/strains, diversity of *Pf*/*Pv*; environmental conditions	Identify epidemiologic and other factors responsible for transmission	Evaluate risk factors in urban vs. rural children; HH risk for infection, asymptomatic gametocytemia and anemia	Examine parasite genetics; resistance and immunity; infection prevalence; vector identification	Obtain prevalence from XS surveys, then focus on urban areas with potential breeding sites
Site-specific references	41–43	44–46	27,47–49	50,51	52–56	57–61	62,63

CSP = circumsporozoite surface protein; EIR = entomological inoculation rate (infectious bites PPY); HH = household; ICEMR = International Centers of Excellence for Malaria Research; M = million; *Pv* = *Plasmodium vivax*; *Pf* = *Plasmodium falciparum*; PPY = per person per year; XS = cross-sectional.

**Table 3:** Summary of the study design, questions, methods, human sampling, and analytical characteristics of each of the seven ICEMR sites conducting research on malaria in urban settings

ICEMR (urban site, region, country)	Amazonia (Mâncio Lima City, Acre State, Brazil)	East Africa (Jinja City, Jinja District, Uganda)	India (Besant Nagar, Chennai City Tamil Nadu State, India)	Latin America (Quibdó City, Chocó Department, Colombia)	Malawi (Communities in Blantyre City, Blantyre District Malawi)	South Asia (Panaji City, Goa State, India)	West Africa (Madina Fall, Thiès City, Thiès Region, Senegal)
Types of epidemiologic studies	Prospective, observational	Incidence and prevalence surveillance	Epidemiologic, experimental, and laboratory-based genetic studies	Descriptive epidemiologic surveillance	Ecologic, environmental and laboratory based	Epidemiologic, experimental, and laboratory based	Community-based longitudinal cohort study
Outcome(s) being measured	Time/GIS pattern by genotype; clonal expansion; vector breeding sites; larval genetic markers to estimate migration	Infection incidence (0.55–0.36/year); prevalance (9.8–6.7%); EIR 4.0–1.8; prevalence of anemia	Prevalance of infection; MOI; genetic diversity of *Pv*; role of sub-patent *Pf* in transmission; vector resting preference; HBI; vectorial capacity and EIR	Prevalance and origin of infection (local or imported); vector presence and predominance; human–vector contact	Urban vs. rural behavioral, SES, environmental risk	MOI, disease severity, genetic variation, drug resistance, *var* gene expression, RBC invasion, human malaria vectors	Infection duration and prevalence; antibodies to vaccine antigens (in study cohort); home visits to evaluate/review symptoms
					Prevalence of parasitemia (including gametocytemia) in healthy subjects		
Exposure(s) or predictor(s) being measured	Environmental: breeding sites; behavior: travel to rural sites and other malaria-endemic areas	LLINs, HH structure, vegetation, temperature, and humidity	Environmental micro-climate, temperature, and humidity (larval and adult vector studies); vector saliva antibodies	Humidity, rainfall, temperature, breeding sites, vector contact by antibodies to vector saliva	Housing, vector proximity, prevention methods	Geographic (migration, travel history), occupational (i.e., migrants, construction workers)	Intensity of vector-to-human transmission; vector breeding sites (GIS)
					Demographic, pregnancy, season		
Study designs being used	PID at government HF	Longitudinal cohort	XS surveys (ACD, and RCD); PCD at clinic and longitudinal cohort designs	XS studies (ACD, RCD) and interventions	Case–control HF + follow-up	PCD at tertiary care government hospital	Cross-sectional, household-based prospective study design
		Community XS/school surveys			XS, HH-based designs		
Sources of subjects	Government HFs	Community HHs	Community HHs	HHs, HFs	HFs, HHs	HFs	Community HHs
					Community HHs		
Selection or sampling method of subjects	Persons seeking malaria diagnosis and treatment at government HFs	Sample of 100 HHs with children + caregiver > 18	Epidemiologic study (random sampling); environmental based (transmission study)	Recruitment of cases at HFs	Any visitor to HF fitting the inclusion criteria	Positives at HFs	Random sample of HHs initially
		Sample of 200 HHs + three schools		Active case detection at two sites with 900 people per site	All HH members		
Definition and determination of urban	Households are considered urban if residents call them urbano	Not predefined	Based on current guidelines from the MoH (UMS of India) and the city of Chennai for the community of Besant Nagar	Urban as defined by a government; examine effects of sociodemographic and geographic factors (including GIS)	Government city limits; multiple definitions and criteria	Defined by Indian government administrative criterion, including agglomerations	City population of 320,000 with 1,000–2,000 *Pf* cases of uncomplicated malaria diagnosed each year at regional clinic
					Random HHs in city		
Age(s) of subject(s)	Persons of all ages	6 months–11 years	≤ 1 to ≤ 70 year old (Eco-Epidemiology Project)	> 2 year old	≤ 15 years old	Subjects ≤ 1–65 years old	Persons of all ages
		Person > 18 years old			All ages		
Sample period	January 2015–January 2016	August 2011–present	XS: December 2013–December 2014	June 2014 (pilot)	April 2012–present	Apr 2012–present	September 2012–present
			Clin: 2013–present		April 2012–present		
			RCD: 2014–present				
			Mosq: 2013–2014				
			Long: February 2013–January 2014				
Sample frequency	5 days/week (Monday–Friday) when HFs open	During illness and at 3 months checkup	Clin, RCD: ∼weekly; XS: quarterly, Long: monthly	Twice yearly	All year, 5 days/week	Daily (one time-point per subject)	Twice yearly XS surveys
					6 weeks × 2/year		
Contextual information or confounders	To be defined	Socioeconomic status, housing	Travel (as a potential confounding factor)	KAP surveys, case investigations (for travel and occupational histories)	KAP, SES, travel (confounders)	Travel, clinical presentation, previous antimalarial treatment	HH conditions (contextual); preventive measures (confounder)
					SES, prevention, ITN, IPTp, location (confounders)		
Unit(s) of analysis	Individual people; HHs parasite: clones breeding sites	Individual people; HHs	Individual people census clusters (XS surveys)	Individuals, HHs, study sites	Individual people, HH, HF, urban–rural environments	Individual people: parasite isolates/clones; vectors at construction sites	Individual people (longitudinal cohort studies), HHs (XS surveys), communities
					Individual people, HH, EA		
Analytical approaches being used	Spatial analysis: human infection genotype clusters near breeding sites	Statistical, modeling and spatial analysis	Spatial analysis; time series	Descriptive and inferential statistics. spatial analysis and statistical modeling	Logistic regression, multilevel, spatial	ICEMR analyses are specialized for each biological question	Initially descriptive, hypotheses based on seasonality, spatial clustering
					Regression, multilevel statistic		

ACD = active case detection; EA = Enumeration Area; GIS = geographic information system; HBI = human biting index; HF = health facility; HH = household; IPTp = intermittent preventive treatment in pregnancy; ITN = insecticide-treated net; KAP = knowledge, attitudes and practices; LLINs = long-lasting insecticidal nets; MoH = Ministry of Health; MOI = multiplicity of infection; ICEMR = International Centers of Excellence for Malaria Research; PCD = passive case detection; PID = passive infection detection; *Pv* = *Plasmodium vivax*; *Pf* = *Plasmodium falciparum*; RBC = red blood cell; RCD = reactive case detection; SES = socioeconomic status; UMS = Urban Malaria Scheme; XS = cross-sectional.

**Table 4:** Summary of the *Plasmodium* and *Anopheles* species present, vector sampling, and laboratory methods at each of the seven ICEMR sites that are investigating malaria in urban settings

ICEMR (urban site, region, country)	Amazonia (Mâncio Lima City, Acre State, Brazil)	East Africa (Jinja City, Jinja District, Uganda)	India (Besant Nagar, Chennai City, Tamil Nadu State, India)	Latin America (Quibdó City, Chocó Department, Colombia)	Malawi (Communities in Blantyre City, Blantyre District, Malawi)	South Asia (Panaji City, Goa State, India)	West Africa (Madina Fall, Thiès City, Thiès Region, Senegal)
*Plasmodium* species	*vivax*, *falciparum* (*malariae*)	*falciparum* (predominant)	*vivax* (predominant), *falciparum*	*falciparum* (61%)	*falciparum*, *malariae*, *ovale*	*vivax* (predominant), *falciparum*	*falciparum*
*Anopheles* species	*triannulatus*, *darlingi*	*gambiae* s.s. (36%), *arabiensis* (64%)	*stephensi*	*nuñeztovari*, *darling*, *albimanus*, *neivai*, *pseudopunctipennis*	*gambiae* s.s., *arabiensis*, *funestus*	*stephensi*	*arabiensis*, *funestus*
Vector sampling	Larvae: dipping	Larvae: dipping	Larvae: dipping	Adults: HLC	Larvae: dipping (outdoor ground)	Adults: CDC light traps in sleeping areas of construction sites	Adults: HLC for biting rates, EIRs
		Adults: CDC light traps	Adult HLC and PSC for biting rates, EIR	Transects from rural to peri-urban and to urban areas	Adults: light traps		PSCs likewise for biting rates, EIRs
Vector testing	ID: morphology, planned: PCR	ID: morphology, PCR	ID: morphology for spp., egg ridge for races or ecological variation	ID: morphology, PCR	ID: morphology, PCR for species	ID: morphology, PCR	ID: PCR
	Blood meal: PCR	Infection: sporozoite CSP by ELISA	Blood meal for host	Infection: sporozoite CSP by ELISA	Infection: sporozoite CSP by ELISA	Genotype: PCR	Infection: sporozoite CSP ELISA
	Infection: sporozoite PCR		Infection: sporozoite CSP by ELISA			Blood meal: PCR	Blood meal: ELISA
						*Plasmodium*: PCR	
Laboratory and field testing	Humans: smears, PCR, nested PCR	Humans: RDT, thick smear microscopy, Hemocue for Hb, immunologic studies	Humans: RDT, Hemocue for Hb	Humans: thick smear, RDT	Humans: slide microscopy, RDT	Humans: RDT, thick and thin smear, PCR, qPCR, sequencing, flow cytometry	Humans: RDT
	Parasite typing based on SNPs or microsatellites		Laboratory: thick and thin smear and PCR	Laboratory: PCR-based parasite species ID	Laboratory: qRT-PCR		Laboratory: thick smear or PCR for diagnosis of human infection

CDC = Centers for Disease Control and Prevention; CSP = circumsporozoite surface protein; EIR = entomologic inoculation rate; ELISA = enzyme-linked immunosorbent assay; Hb = hemoglobin; HF = health facility; HLC = human landing catches; ICEMR = International Centers of Excellence for Malaria Research; PSC = pyrethroid spray capture; qRT-PCR = quantitative reverse transcription polymerase chain reaction; RDT = rapid diagnostic test; SNP = single nucleotide polymorphism test.

**Table 5:** Research questions of common interest across the ICEMR network at urban sites in South America, Asia, and sub-Saharan Africa

Evaluation of malaria in urban areas	Malaria in urban areas ranges from foci of intense transmission to obvious importations in travelers returning from highly endemic areas
	Because the intensity of transmission is typically lower in urban than rural areas, proof of urban malaria transmission is uncommon
	This inadvertently means that the term “urban malaria” is often applied empirically to all persons whose malaria was diagnosed in urban areas
	Although difficult, the proof of transmission (or the lack of transmission) in urban areas is an essential priority for malarial control
	This is because proof of transmission simultaneously provides both new information and potential malaria control strategies
Descriptive epidemiology	What are the age distributions of malarial infection (parasitemia, positive smears) and disease (uncomplicated and complicated/severe malaria)?
	Is there evidence that children, adults, or others are protected from (or at increased risk of) infection or disease?
Seasonality	Does the prevalence of infection (parasitemia) decrease during the dry season and increase with the return of seasonal rains?
	How are seasonal patterns such as rainfall related to the incidence of disease?
	When does the incidence of malarial disease peak in relation to the intensity of transmission and the peak prevalence of infection?
Length of residence	The effects of prolonged residence in this or other malaria-endemic areas
	Do persons who have lived in this or other malaria-endemic areas for ≥ 10 years acquire either the semi-immune state (protection against serious disease) or sterile immunity (protection against both infection and disease)?
Entomologic factors	What vectors are present at different study sites during the different times (seasons) of the year?
	How do their biting rates and EIRs relate to the frequency of human infection and disease?
	Are these characteristics in more urban settings similar to or different from what has been found in rural areas?
Complex malaria	Why are infections with more than one parasite genotype most common with *Plasmodium falciparum* in sub-Saharan Africa?
	Conversely, why are infections with more than one parasite species (e.g., *P. falciparum* and *Plasmodium vivax*) more common in India, other parts of Asia, and South America?
	Why are *Plasmodium ovale* and *Plasmodium malariae* infections much less common than *P. falciparum* in Africa?

EIRs = entomologic inoculation rates; ICEMR = International Centers of Excellence for Malaria Research.
